# FRAILTY STATUS, COGNITIVE IMPAIRMENT, AND THE EFFECTS OF INTENSIVE BLOOD PRESSURE CONTROL

**DOI:** 10.1093/geroni/igz038.2945

**Published:** 2019-11-08

**Authors:** Kathryn E Callahan, Maryjo Cleveland, Mark Supiano, Jeff Williamson, Nicholas M Pajewski

**Affiliations:** 1 Wake Forest School of Medicine, Winston-Salem, North Carolina, United States; 2 University of Utah School of Medicine Center on Aging, Salt Lake City, Utah, United States

## Abstract

Background: Frailty associates with cognitive decline and incident dementia in older adults. The Systolic Blood Pressure Intervention Trial (SPRINT) has highlighted blood pressure (BP) control as a potentially modifiable risk factor for cognitive impairment. Using data from SPRINT, we explore whether frailty status, based on a frailty index (FI), prospectively associates with mild cognitive impairment (MCI) and dementia, and whether the effect of intensive BP control on these outcomes varies by frailty status. Methods: SPRINT randomized participants to either to an systolic BP goal of <120 mmHg (intensive treatment) or a goal of <140 mmHg (standard treatment). We used Cox regression to model the association of the FI with MCI and dementia, and to conduct subgroup analyses by frailty status for the effect of intensive treatment. Results: We include 9307 participants, with the majority categorized as pre-frail (0.100.21, 38.0%). Adjusting for age, sex, race/ethnicity, education, and treatment group, a 0.1 increase in the FI was associated with increased risk for MCI (Hazard Ratio (HR) = 1.42, 95% CI: 1.29, 1.58) and dementia (HR = 1.80, 95% CI: 1.56, 2.08). There was weak evidence of an interaction between frailty status and intensive treatment for the composite outcome of MCI and dementia (p=0.03), with a beneficial effect of intensive treatment in pre-frail participants (HR=0.71 95% CI: 0.58, 0.89), and a largely null effect in frail participants (HR=0.98, 95% CI: 0.82, 1.18). Conclusions: Frailty status may modify the effect of intensive BP control on MCI and dementia.

